# Research on the Translation and Implementation of Stepping On in Three Wisconsin Communities

**DOI:** 10.3389/fpubh.2017.00128

**Published:** 2017-06-12

**Authors:** Amy E. Schlotthauer, Jane E. Mahoney, Ann L. Christiansen, Vicki L. Gobel, Peter Layde, Valeree Lecey, Karin A. Mack, Terry Shea, Lindy Clemson

**Affiliations:** ^1^Injury Research Center, Medical College of Wisconsin, Milwaukee, WI, United States; ^2^Department of Medicine, University of Wisconsin School of Medicine and Public Health, Madison, WI, United States; ^3^Greater Wisconsin Agency on Aging Resources, Inc., Madison, WI, United States; ^4^National Center for Injury Prevention and Control, Centers for Disease Control and Prevention, Atlanta, GA, United States; ^5^University of Wisconsin Hospital and Clinics, Madison, WI, United States; ^6^University of Sydney, Sydney, NSW, Australia

**Keywords:** *Stepping On*, falls prevention, dissemination, implementation, preventing falls

## Abstract

**Objective:**

Falls are a leading cause of injury death. *Stepping On* is a fall prevention program developed in Australia and shown to reduce falls by up to 31%. The original program was implemented in a community setting, by an occupational therapist, and included a home visit. The purpose of this study was to examine aspects of the translation and implementation of *Stepping On* in three community settings in Wisconsin.

**Methods:**

The investigative team identified four research questions to understand the spread and use of the program, as well as to determine whether critical components of the program could be modified to maximize use in community practice. The team evaluated program uptake, participant reach, program feasibility, program acceptability, and program fidelity by varying the implementation setting and components of *Stepping On*. Implementation setting included type of host organization, rural versus urban location, health versus non-health background of leaders, and whether a phone call could replace the home visit. A mixed methodology of surveys and interviews completed by site managers, leaders, guest experts, participants, and content expert observations for program fidelity during classes was used.

**Results:**

The study identified implementation challenges that varied by setting, including securing a physical therapist for the class and needing more time to recruit participants. There were no implementation differences between rural and urban locations. Potential differences emerged in program fidelity between health and non-health professional leaders, although fidelity was high overall with both. Home visits identified more home hazards than did phone calls and were perceived as of greater benefit to participants, but at 1 year no differences were apparent in uptake of strategies discussed in home versus phone visits.

**Conclusion:**

Adaptations to the program to increase implementation include using a leader who is a non-health professional, and omitting the home visit. Our research demonstrated that a non-health professional leader can conduct *Stepping On* with adequate fidelity, however non-health professional leaders may benefit from increased training in certain aspects of *Stepping On*. A phone call may be substituted for the home visit, although short-term benefits are greater with the home visit.

## Introduction

Unintentional falls have been the leading cause of injury death in adults aged 65 years and older from 1999 to 2014 (1). The cost of fatal fall injuries in 2010 totaled $2 trillion among older adults (2). While evidence-based fall prevention interventions exist, they have not been widely implemented in local communities by public health, human service, and health-care practitioners (3–5). Research on implementation can provide insight into the barriers and facilitators that organizations experience in trying to adopt and implement these programs in community settings in order to maximize the spread and implementation of science-based fall prevention interventions (6–11). This type of research can also identify key programmatic elements that are critical to maintaining fidelity to the original intervention, thus maintaining program effectiveness (9). Likewise, implementation research can identify adaptations that local organizations may wish to make to facilitate adoption, and test such adaptations to ensure that fidelity and effectiveness are maintained (9–11). Implementation research can elucidate the impact of the programs on the individuals served to determine who is most likely to be reached in a community by the program and whether the program continues to be effective at preventing falls outside the context of the original research study (6). This information is critical to informing the packaging, marketing, and distribution of a given program so that communities know what programs are appropriate and feasible to implement for their populations (6).

*Stepping On* is a multifaceted fall prevention program that uses a series of small group sessions followed by a home visit and a 3-month booster session to teach fall prevention strategies to community-dwelling older adults, improve fall self-efficacy, encourage behavioral change, and reduce falls (12). In a randomized trial with community-dwelling adults in Australia, *Stepping On* was shown to reduce falls by 31% (12). It has also been shown to provide a positive return on investment (13).

The purpose of this study was to examine aspects of the translation and implementation of *Stepping On* in three community settings in Wisconsin. Based on prior work and knowledge of the program, the investigative team identified several areas to study, to understand the spread and use of the program, as well as to determine whether critical components of the program could be modified to maximize use by communities (14–16). Specifically, the investigative team sought to evaluate five common implementation research outcomes (program uptake, participant reach, program feasibility, program acceptability, and program fidelity) by varying the implementation setting and constructs of *Stepping On*.

## Materials and Methods

Six *Stepping On* workshops were held in total across three community sites in Wisconsin (Table 1).

**Table 1:** Implementation setting, evaluation of *Stepping On* in three Wisconsin communities.

Program site		Urban versus rural	Class leader background	Format (home visit versus phone call)	Participants (*n*)
1.	Independent Living Retirement Community (ILRC)		Health degree		9
2.	ILRC		Non-health degree		10
3.	Parks and Recreation Center	Urban		Home visit	11
4.	Parks and Recreation Center	Urban		Phone call	12
5.	Parish nurse	Rural		Home visit	10
6.	Parish nurse	Rural		Phone call	11

*Independent Living Retirement Community (ILRC)* — Two workshops were held at an ILRC located in an urban area in Southeastern Wisconsin. The leader of one workshop was from a health background (ILRC RN), while the leader of the other had a non-health professional background (ILRC senior service manager). Both leaders had implemented one workshop as a pilot and had received feedback based on a content expert’s fidelity observations. Both workshops utilized home visits. The total number of participants enrolled at this site was 19.

*Parks and Recreation Center* — Two workshops were held at a Parks and Recreation Center located in an urban area in Southeastern Wisconsin. The leader of these workshops was a speech therapist by training, and a fitness expert by current occupation. The first workshop at this site utilized home visits while the second utilized phone calls from the leader in lieu of home visits. The total number of participants enrolled at this site was 23.

*Parish Nurse Program* — Parish Nurse Program Two workshops were held through the parish nurse program in a small town in a rural area of Southeastern Wisconsin. The leader of these workshops was a parish nurse (RN). The first workshop at this site received a home visit while the second received a phone call from the leader in lieu of a home visit. The total number of participants enrolled in this site was 21.

### Evaluation Measures

Four research questions guided this evaluation. The implementation metrics of interest and methodology to examine each differed and are described below by question, and summarized in Table 2.

**Table 2:** Methodological details for study research questions.

Question	Rationale for question	Comparison	Implementation metric(s) of interest	Specific questions
Who could serve as Stepping On leader?	Original Stepping On leader was health professional; fidelity may be worse with non-health professional	Leader with health degree	Implementation fidelity	Is implementation fidelity decreased with a workshop leader without a health degree, compared to a leader with a health degree?
		Leader without health degree		

How does implementation of program vary across sites?	Program has not been implemented in Independent Living Center or Parish Nurse Program; it is unknown if there are barriers to feasibility, uptake, and participant reach in these settings	Independent Living Retirement Community	Participant reach	How does participant reach vary by implementation site?
		Parks and Recreation Center	Program feasibility	Will program feasibility differ across sites?
		Parish Nurse Program	Program uptake	Will program uptake differ across sites?

How does implementation vary between rural versus urban sites?	Rural sites may have more difficulty implementing the program due to less access to physical therapists	Rural site (1)	Participant reach	How does participant reach vary between rural versus urban sites?
		Urban sites (2)	Program feasibility	Will program feasibility factors differ between rural and urban sites?

Can a phone call be substituted for a home visit?	Home visit may be more difficult to implement	Program implementation with home visit	Program acceptability	Are phone calls more acceptable to leaders and site managers, compared to home visits?
		Program implementation with phone call		
			Program fidelity	Is program fidelity decreased by phone call compared to home visit?
			Participant uptake at 1 year	Is participant uptake at 1 year decreased by phone call compared to home visit?

### Question 1: Who Could Serve As a *Stepping On* Leader?

The original program manual suggested the following health professionals could be *Stepping On* Leaders: “occupational therapist, physiotherapist, and other health professional and health promotion worker in the area of falls-promotion with older people” (17). Preliminary experience indicated that limiting who could lead the program to these professions could create an organizational barrier to implementation in a community setting. The investigative team sought to determine if having a non-health professional leader compromised program fidelity. The implementation metrics of interest include observing the program fidelity for the health professional as compared to the non-health professional.

Leader fidelity to the program was measured for the two workshops (one facilitated by a health professional, one by a non-health professional) held at the ILRC by expert observation using a checklist during four of the seven workshop sessions. The observer was a retired nurse (RN) who had served as a peer leader for several previous *Stepping On* workshops, and a Co-Trainer for several previous *Stepping On* Leader trainings. The checklist was developed based on essential elements of the program determined by Delphi consensus of an international expert panel (14). Fidelity observations were captured by both occurrence and quality. The observer marked “occurred” or “did not occur” for the listed key elements. Additionally, the leader was given a quality rating: A—excellent; B—very satisfactory; C—satisfactory; D—not satisfactory; F—not done at all. The quality ratings were translated to numerical scores for analysis: A—4 points, B—3 points, C—2 points, D—1 point, F—0 points. The observer was asked to comment on all items that were not satisfactory. Space was provided at the end of the checklist for the observer to add any additional comments. The items on the fidelity scales were reviewed by two of the authors (JM, LC), both experts in fall prevention and in the *Stepping On* program, and the following subscales were created: program occurrence, program quality, exercise occurrence, exercise quality, leader quality as a facilitator and adult educator, peer leader quality, physical therapist (PT) elements—occurrence, and, PT elements—quality (15). PT elements were judged based on the activities that the invited PT led in Sessions 1, 2, and 6. Occurrence subscales produced a percent occurred (out of total number of items in the subscale) as a final numerical score. Quality subscales produced a final mean score. Qualitative analysis examined differences in checklist item scores as well as mean score comparing the two types of leaders. Observer’s comments were examined for themes by two independent coders (18).

### Question 2: Are There Differences in the Implementation of the Program across Differing Sites?

The original program called for implementation in a suitable community-based venue that is easily accessible to the public. The investigative team identified types of community-based settings, which had potential to implement *Stepping On* and reach large numbers of older adults at risk for falls. Within each of these settings, however, a number of organizational barriers may exist which could prevent successful uptake and implementation. The investigative team studied the implementation in an ILRC, a park and recreation center, and a parish nurse program to examine the implementation metrics of participant reach, program feasibility, and program uptake.

In-depth qualitative interviews of leaders and site managers after workshop completion at all sites asked about barriers and uptake, acceptability, adaptability, and feasibility of implementing the program at the site. Qualitative protocols were developed by the study team. Interviews included semi-structured and open-ended questions. Additionally, sites provided basic demographic information (age, gender, race, ethnicity as available) regarding clientele served at their site. This information was used to examine the extent to which workshop participants represented the demographics of the clientele at each site. Interview transcripts were hand coded and examined for themes by two independent coders (18).

### Question 3: Are There Differences in the Implementation of the Program between Rural versus Urban Sites?

One concern for the investigators was that rural areas may not have access to some of the experts required to provide guest information sessions within the program. This included a PT to contribute to three of the sessions. Additional, lack of transportation alternatives may impede reach to older adults in rural areas. We conducted rural versus urban analyses to examine implementation metrics of participant reach, and program feasibility.

Rural and urban were defined based on location of the workshop, using the State of Wisconsin Bureau of Aging and Long Term Care classification of rurality (19). Locations were considered rural if they were in a county that had fewer than 20 people 60 years of age or older per square mile, and if they were not part of a federally designated Metropolitan Statistical Area (19). We examined geographic reach based on one-way distance traveled by participants and guest experts as well as the representativeness of participant reach in relation to the catchment areas of the site. Mean and SD of miles traveled were calculated and compared using *t*-tests with STATA v.12.

Methods of recruitment of participants depended on the site. The parks and recreation center had waiting lists for the class and had no problems recruiting participants. The *Stepping On* workshop was added to the list of offerings and participants signed up for it. The ILRC did not hold workshops of any type and consequently, the site manager made phone calls to offer the program to residents. The parish nurse who led the workshop recruited participants at the third site through advertising in the church bulletin and through personal invitation to clients who she felt might benefit.

### Question 4: Can a Phone Call Be Substituted for a Home Visit?

The home visit is one component called for by the original program to assist with, reinforce, and support follow-through of fall prevention strategies and activities, including exercise, and supplement participant assessments of fall hazards and assist with remediation of those home hazards (20). In resource constrained areas, a phone call is the more economical option. The investigative team was concerned that while a phone call may be more acceptable to leaders and site managers, implementation metrics of fidelity and participant uptake at 1 year may be adversely affected by replacing the home visit with a phone call.

Three tools were developed by the study team to examine the question of home visit versus phone call within the parks and recreation and parish nurse sites. These tools were based on the home visit questionnaires designed for the original *Stepping On* study. The first tool was completed by the leader immediately after each home visit or phone call with a participant. This tool contained structured and open-ended questions regarding the discussion that occurred between participant and leader as part of the home visit or phone call. Questions asked about content of discussions and numbers of recommendations about three target areas that the manual suggests are discussed on the home visit: strategies the participant uses for fall prevention, performance of workshop exercises at home, and home hazards identified. Qualitative analysis of key themes was done by two independent reviewers.

The second tool was a qualitative and semi-quantitative survey completed 1 year after the home visit or phone call. Research staff contacted the participants by phone and asked them to assess the extent to which they had followed through on items and strategies discussed in the home visit/phone call. Frequency tabulations and Fisher’s exact tests were conducted for differences in performance on both tools by leader by site for the parish nurse and parks and recreation sites. Qualitative comments were reviewed for key themes.

A third tool was a questionnaire mailed to participants within 2 weeks of the home visit/phone call. It asked participants to rate the perceived benefit of the encounter overall, and of elements of the encounter considered as key elements based on the Delphi consensus using a scale of 1 to 10, with 10 being the most benefit. Participants were asked to say if an element occurred as well as rate their perception of usefulness of that element. Frequency tabulations and Fisher’s exact tests were conducted and qualitative comments were reviewed for key themes.

This study was reviewed and approved by the Institutional Review Board at the University of Wisconsin-Madison School of Medicine and Public Health. All informants (leaders, peer leaders, guest experts, site manager, and participants) gave informed consent to answer questionnaires and/or be interviewed.

## Results

### Question 1: Who Could Serve As a *Stepping On* Leader?

The nine fidelity subscale scores for each leader are presented in Table 3. For both leaders, key program activities or elements occurred over 80% of the time. Quality scores on subscales for both leaders were in the satisfactory to very satisfactory range. The health professional leader scored higher on five of the subscales; lower on two of the subscales and the same on two subscales compared to the non-health professional leader. When looking at specific items on the fidelity tools, the non-health professional had a score of not satisfactory or not done/did not occur on at least one occasion in the following: linking exercises to how they prevent falls or improve function, demonstrating knowledge of falls prevention topics, correcting or reinforcing the guest expert PT to ensure activities aligned with the manual, and using the program’s problem-solving framework during the session to maximize adult learning. Both the health professional and the non-health professional had difficulty with time management, often running out of time for class components. Neither used weights in exercise practice during the sessions. The fidelity observer commented that both leaders improved over time with regard to being more facilitator-like in style (versus teacher like), a key element according to the Delphi process. In summary, while both leaders achieved satisfactory quality with delivery, the non-health professional showed lapses to fidelity in four critical areas of the *Stepping On* program.

**Table 3:** Fidelity subscales by leader type (health professional versus non-health professional).

Fidelity subscale	Health professional leader score (*n* = 1)	Non-health professional leader score (*n* = 1)
**Occurrence (0–100 scale)**		
Program occurrence	83.9%	87.5%
Exercise occurrence	96.3%	96.3%
Physical therapy occurrence	97.5%	97.4%
**Quality (0–4 scale)**		
Program quality	2.66	2.77
Exercise quality	3.43	3.18
Exercise quality subscale	3.39	3.14
Global leader quality	3.35	2.69
Peer leader quality	3.50	3.50
Physical therapy quality	3.38	3.12

*Shading indicates higher fidelity score*.

### Question 2: Are There Differences in the Implementation of the Program across Differing Sites?

The three sites had different experiences in implementing the program. Scheduling the PT was noted as an area that could be burdensome for the leader and/or site manager in two of the three sites, in particular for the first time the site held a workshop. The parish nurse site paid the PT to participate. “*I think if we could not pay the PT, it would be very difficult, if not impossible to get one with the shortage of PTs at this time*.” The other sites did not pay the PT. The parks and recreation site also had difficulty finding a PT, largely due to timing. The site manager at the senior center noted that “*PTs book schedules months in advance so in order to get a PT to commit to all classes, you would have to do this months in advance*.”

Leaders and site managers noted that program tasks that were burdensome when leading their first class were not as burdensome with the second. Leaders noted that pacing the session, their preparation of the guest experts, facilitating exercises and the progression of exercises all improved with the second class. Of note, experts, all of whom were volunteers except for the one paid PT, who were difficult to schedule for the first class were not difficult to schedule for the second class, likely due to the fact that the same experts who were used in class one were used in class two.

Overall, implementation of *Stepping On* imposed the largest burden on the ILRC. Staff members were not given additional time or a reduction in workload to offset the time spent on the workshops. Leaders commented “*it was hard to balance job with class responsibilities*.” The research placed additional demands on the program staff, particularly around when workshops were offered. Workshops were held according to the research schedule and not when the facility would normally have offered them. The site manager noted that recruitment was “harder” at the ILRC and involved a lot of time making phone calls. Conversely, the *Stepping On* mission and workshops were aligned with the mission of both the parks and recreation and parish nurse programs. The parish nurse noted that it was difficult to get the recommended number of participants (8–10) sharing the same “*educational interest, same schedule and same level on interest [in the program]*”; however, word of mouth made recruitment easier for the second workshop at the parks and recreation center and parish nurse sites. Although all sites said they would host a *Stepping On* workshop again after the end of the research study, this happened at only the parks and recreation and the parish nurse sites. Further workshops were not held at the ILRC.

#### Implementation in Different Community Sites

The enrolled populations differed across the three sites (Table 4). The gender composition of the ILRC participants (82% female) was representative of the gender composition of the ILRC population (79% female). The racial background of the ILRC population was 95% White and 5% Black. The racial background of the ILRC workshop participants was similar: 97% White, 3% Black.

**Table 4:** Characteristics of Stepping On workshop participants in three community sites in Wisconsin.

	Independent Living Retirement Community (ILRC) workshop participants	ILRC population	Parks and Recreation Center workshop participants	Parks and Recreation Center population^a^	Parish nurse workshop participants	Parish nurse population^a^
Race/ethnicity^b^	97% W	95% W	100% W	79% W	100% W	91% W
	3% B	5% B		21% H		9% H
% Female	82	79	91	85	90	60
Age (mean)	83.5		76.5		78.1	
% Use assistive device	52		17		19	
% With less than high school education	23		4		5	
# of Falls in previous year (mean)	1.28		0.87		0.52	

*^a^Populations based on the city of site location*.

*^b^W, white; B, black; H, hispanic*.

The gender composition of the parks and recreation center participants (91% female) and of the population attending the center as a whole (85% female) was predominately female. The population of the city where the parks and recreation center was located is 79.0% Caucasian/White and 21% Hispanic. However, all workshop participants were White.

The gender composition of the parish nurse workshops (90% female) was not representative of the gender composition of the older adult congregation population (60% female). The population of the city where the church was located is 91.0% Caucasian/White and 9% Hispanic, with much of the Hispanic community attending the church sponsoring the workshop. However, all workshop participants were White. Per the site manager, Hispanics attending that church do not participate in group activities with non-Hispanics. In addition, many Hispanics aged 65 and over in the congregation do not speak English, and many are not literate.

Workshop attendees at the ILRC were older, all from senior centers, and had less formal education than did attendees from other sites. A higher percentage used assistive devices and there was a higher average number of falls in the 6 months prior in this group. The characteristics of participants at the parks and recreation and parish nurse programs were similar.

### Question 3: Are There Differences in the Implementation of the Program between Rural versus Urban Sites?

There was no significant variation in one-way distance traveled by participants attending workshops in the rural site (parish nurse) compared to the suburban (parks and recreation center), despite the fact that the parish draws from a 20 miles radius for the congregation. Participants in the parish nurse workshop came from within a 5 miles radius from the church. The recreation center draws from a 5 miles radius for 95% of its participant population, and the participants in the *Stepping On* workshops did as well. The reach in the rural setting was no different than the reach in suburban (median one-way distance traveled of 2.0 miles, range 0–4 miles, for rural setting versus median of 2.0 miles, range 0–55 miles, for suburban, *p* = 0.24). Participants at the ILRC lived at the apartment complex and thus did not travel to attend workshops.

Guest experts had to travel significantly more miles to attend workshops in the rural site compared to the other sites. Non-PT experts in rural areas traveled significantly more miles (mean of 19.5 miles, SD 6.7) to get to the class location than non-PT experts in urban areas (mean of 1.3 miles, SD 0.6, *p* = 0.0111). However, the experts in rural areas did not report that their travel was burdensome. There were no rural/urban differences in self-reported burden for travel. There were no rural/urban differences in terms of PT miles traveled and burden.

### Question 4: Can a Phone Call Be Substituted for a Home Visit?

The differences in the home visit versus phone call varied by leader. With the health professional at the parish nurse program, there were no significant differences between the home visit and phone call in terms of strategies, exercises, and home hazards with the exception of one. More participants demonstrated the exercises for the leader in the home visit group (75%) as compared to the phone call group (0%, *p* = 0.007). With the non-health professional at the ILRC, there were several significant differences between the groups with strategies, exercises, and number of home hazards identified (Table 5).

**Table 5:** Significant home visit versus phone call programmatic differences with non-health professional leader (n = 44).

	Home visit (% Yes)	Phone call (% Yes)	*p*-Value
As a result of your meeting did the participant identify any plans to better integrate strategies into everyday life?	57	0	0.026
As a result of your meeting did the participant make a plan for the next steps regarding exercise?	100	63	0.082
Did the participant demonstrate how they do the exercises?	86	0	NA
Did you (the leader) demonstrate any corrections or advancements of the exercises for the participant?	67	0	0.009
Did the participant identify a second hazard in or around the house?	56	0	0.029

*NA (not applicable) as the participants on the phone did not demonstrate exercises.

Comfort and previous experience conducting home visits played a role for leaders. Home visits were part of existing programming at two of the three sites (ILRC and parish nurse program), thus leaders had done home visits before and were comfortable with the idea. Participants, having received home visits before, were also comfortable receiving home visits as part of the program. In contrast, the parks and recreation center staff did not do home visits as part of any programming and leaders and the site manager noted that some participants were uncomfortable with the idea of the leader coming to the home.

The home visit placed a burden on two of the three organizations (ILRC and parks and recreation center). The parks and recreation center felt the home visit was time consuming and imposed a travel burden. The ILRC staff found the preparation for home visits more burdensome than expected. All leaders from all three sites preferred the home visit to the phone call, even with the additional burden. “*You cannot see their body language [with a phone call],”* the ILRC leader commented. Leaders also commented that it was impossible to observe and correct exercises over the phone and felt that participants may be more honest at a home visit as compared to the phone.

Two weeks after the home visit or phone call, participants receiving the phone call (*n* = 17) had an overall perception of that encounter as less helpful compared to participants receiving the home visit (7.0 versus 8.8 on a scale of 0 = not helpful at all to 10 = extremely helpful; *p* = 0.023). Of the six elements considered key to the home visit or phone call, participants reported a significantly greater number of these elements occurring with the home visit versus the phone call (mean 5.25 versus 4.18; *p* = 0.026). In particular, referrals were made less often in the phone call versus home visit, (19% of phone calls versus 75% of home visits (*p* = 0.004)), and participants viewed the referrals as less helpful when made by phone compared to in-person. At 1 year follow-up with participants, comparing those receiving a home visit with those receiving a phone call, there were no differences in the number of actions taken in response to the home visit/phone call discussion of strategies, exercises, and home hazard remediation.

## Discussion

This implementation research study examined several key questions regarding program adaptation and fidelity of *Stepping On* in the United States in order to determine how best to maximize spread and implementation of this evidence-based program. Findings demonstrated that overall, a health professional and non-health professional were similar in fidelity by their second workshop. Nevertheless, subtle differences emerged that may be due to background, particularly in the area of fidelity, a key component of implementation research. The non-health professional showed lapses of fidelity in a few key areas: linking exercises to function and how they prevent falls, using the preventive framework (a set of specific prompts used in the program to facilitate discussion and action), and guiding the PT to ensure that exercise is practiced during the therapist’s session and participants get the opportunity to practice mobility activities outdoors. These fidelity lapses may be due to individual rather than educational background differences. Teacher/leader training has been demonstrated to be a key component of successfully implementing interventions in a community setting (7). However, caution should be exercised when training non-health professionals to ensure they understand and master key clinical domains such as those related to exercise and falls and to program facilitation.

Differences in program implementation emerged across the sites, highlighting key implementation science areas such as how communities can make informed decisions about whether this program is a match for their organization in terms of participant reach, program feasibility, and program uptake (10, 11). The program was most easily implemented at the parks and recreation center, where site managers and leaders were used to providing exercise classes. At the ILRC, the program was difficult to implement given multiple competing obligations on the leaders’ and site managers’ time. Specifically, they were not used to providing workshops and did not have staff to easily accommodate the demands of *Stepping On*. At the parish nurse site, it was not difficult to get parishioners to join, and the nurse was comfortable in the leader role. Some similarities occurred across all sites. At all sites, up-front time of several months was required to recruit the guest expert PT for the first workshop. Other experts were easier to recruit and did not require as much advance notice. Recruiting guest experts was easier for workshops subsequent to the first as all experts returned. Leaders became more familiar with workshop material and were more “facilitator-like” by their second workshop.

Participant reach is another key aspect of implementation science (10, 11). Our findings demonstrate that regardless of whether a workshop is held in a rural or a suburban location, attendees tend to come from a radius of 5 miles or less. No significant differences emerged in difficulty of engaging guest experts or burden of travel for guest experts comparing rural or urban sites. Thus, *Stepping On* appears equally well suited for small town and urban areas, but it should not be expected to draw older adults living further than 5 miles from the workshop site.

Program acceptability, fidelity, and uptake were examined by the question of whether or not a phone call could be substituted for the home visit. The home visit required substantially more time than did the phone call, and overall, participants and leaders tended to perceive it as more beneficial. Leaders were able to demonstrate exercises and provide referrals more often at home visits. The discussion of barriers to implementing strategies tended to be more in-depth at home visits. By 1 year, however, there were no obvious differences in participant implementation of new strategies or extent to which exercise was continued. Thus, based on study findings, the authors conclude that it is unclear if the home visit is essential to the success of the program, or if the benefit outweighs the cost. Community programs may want to consider other factors when making a decision on this aspect of the program (e.g., being able to connect the client with other services if needed, for example, referring client to a local fire department if the leader notices that the home does not have a working smoke detector).

This study has a number of limitations. First, small sample sizes precluded examination of differences in participant outcomes of falls as well as a rigorous quantitative analysis at the site level. Second, participation at the participant level in the research study was often affected by the season. In the winter months, many older adults leave Wisconsin for warmer climates. This affected attendance at the parish nurse and parks and recreation sites, but not at the ILRC given that the residents lived at the class location. Third, data on reach were limited to specific demographic data that the sites collected for their target population (gender, race/ethnicity). Thus, for example, we do not know if the age distributions of workshop participants represented the age distribution of the catchment population.

The goal for this research study was to understand how, when, by whom, and under what circumstances *Stepping On* was implemented at the frontline community level in order to inform future program guidance and the best formats for delivering programs. This research was essential to successful implementation and widespread replication of *Stepping On*. These findings have already informed the third North American edition of *Stepping On*, the training program for *Stepping On* leaders, and the *Stepping On* Implementation guide^1^ for sites in the United States. Findings from this research have led to a modification of prerequisites for being a *Stepping On* leader. Rather than limiting training to those with physical therapy, occupational therapy, nursing, or similar health professional background, the program disseminators now offer training to social workers, fitness experts, and health educators who have some prior training and work experience with older adults and experience with group facilitation. Further, the program has been modified so that while a home visit is strongly recommended, it is no longer required. For those interested in implementing *Stepping On*, the disseminators stress the fact that although guest experts can be recruited on a volunteer basis, sufficient lead time is needed, particularly for PTs. Disseminators also stress that to maximize reach, the workshop needs to be implemented at a location within 5 miles of where participants live.

In conclusion, the types of findings in this implementation research study are invaluable to the successful spread of *Stepping On*. Only by testing implementation in a community versus laboratory setting are we able to determine the “how, when, by whom and under what circumstances” (6–11). Programs such as *Stepping On*,^2^ implemented in community settings, can help safeguard older Americans so they stay healthy, active, and independent longer.

## Ethics Statement

This study was carried out in accordance with the recommendations of Institutional Review board at the University of Wisconsin-Madison School of Medicine and Public Health with written informed consent from all subjects. All subjects gave written informed consent in accordance with the Declaration of Helsinki. The protocol was approved by the Institutional Review board at the University of Wisconsin-Madison School of Medicine and Public Health.

## Author Contributions

AS assisted with study design, development of data collection tools, conduct of the research, data analysis, and was responsible for manuscript preparation. JM was responsible for conceptualizing the theoretical and empirical formulations of the research project, literature review, study protocol and design, and analyzing and interpreting data, and provided feedback on draft versions of the paper. AC, PL, KM, and TS assisted with design of the research study, development of data collection tools, and analysis of results and provided feedback on draft versions of the paper. LC assisted with conceptualizing the theoretical and empirical formulations of the research project, study protocol and design, analysis and interpretation of data, and provided feedback on results. VG assisted with development of data collection tools, collection of data, and analysis of results, and provided feedback on draft versions of the manuscript. VL assisted with development of data collection tools and provided feedback on draft versions of the manuscript.

## Conflict of Interest Statement

JM and LC are co-authors on the Stepping On Leader Manual, Third North American Edition, Freiburg Press, Cedar Falls, IA; 2011. The other authors declare no conflict of interest.

## Footnotes

^1^https://wihealthyaging.org/_data/files/SO_materials/Stepping-On-Manual_10-17-2013.pdf.

^2^https://wihealthyaging.org/stepping-on.
